# Evaluation and prediction of water conservation of the Yellow river basin in Sichuan Province, China, based on Google Earth Engine and CA-Markov

**DOI:** 10.1016/j.heliyon.2023.e17903

**Published:** 2023-07-01

**Authors:** Zhichong Yang, Xiaoai Dai, Heng Lu, Chao Liu, Ruihua Nie, Min Zhang, Lei Ma, Naiwen Li, Tiegang Liu, Yuxin He, Zhengli Yang, Ge Qu, Weile Li, Youlin Wang

**Affiliations:** a College of Earth Science, Chengdu University of Technology, Chengdu 610059, China; b State Key Laboratory of Hydraulics and Mountain River Engineering, Sichuan University, Chengdu 610065, China; c College of Hydraulic and Hydroelectric Engineering, Sichuan University, Chengdu 610065, China; d School of Environmental and Geographical Sciences, Shanghai Normal University, Shanghai 200234, China; e School of Geography and Ocean Science, Nanjing University, Nanjing 210093, China; f State Key Laboratory of Geohazard Prevention and Geoenvironment Protection, Chengdu University of Technology, Chengdu, 610059, China; g Northwest Engineering Corporation Limited, Xi'an 710065, China

**Keywords:** Water conservation, Yellow river, Google Earth Engine, CA-Markov, Spatio-temporal analysis

## Abstract

The Yellow River Basin in China has the world's most serious soil erosion problem. The Yellow River Basin in Sichuan Province (YRS), as the upper reaches of the Yellow River, and its water conservation (WC) capacity greatly affects the ecological environment of the downstream basin. In recent years, YRS has received more and more attention, and numerous policies have been developed to improve local WC. However, there is a vacancy in the long-term research of WC in the YRS due to the lack of in-situ data. This study quantitatively evaluated the WC of YRS from 2001 to 2020 through Google Earth Engine (GEE) and analyzed the spatio-temporal variations of WC and land cover (LC). CA-Markov predicted the LC and WC in 2025 under three scenarios to assess the contribution of different scenarios to WC. The WC in YRS fluctuated from 1.93 to 6.77 billion m^3^. The climate is the dominant factor of WC change, but the effect of LC on WC is also evident. The WC capacity increases with vegetation coverage and height. The WC capacity of forests per km^2^ exceeds 600 mm, while that of grasslands is about 250 mm, and barren can cause around 300 mm of WC loss. In 2025, the WC in YRS may exceed 7.5 billion m^3^, but the past ecological management mode should be transformed. Improving the quality of land use and converting grasslands to forests is better than reducing cropland to improve WC.

## 1 Introduction

The Yellow River is one of the longest rivers in the world and the second longest river in China. China's “Mother River”, it feeds 12% of the population and supplies 15% of the irrigated area in China [1]. Due to the fragile ecological environment around it [2], the Yellow River has been the focus of environmental management throughout history. The Yellow River carries over one billion tons of sediment annually, making an area of 795,000 km^2^ in the nine provinces it flows through under threat of soil erosion [1]. The soil erosion area in China is 3.56 billion km^2^, accounting for 37% of the total land area. It is one of the most serious ecological and environmental problems in China. YRS belongs to the Yellow River source region and is the important ecological corridor between the Qinghai-Tibet Plateau and Loess Plateau. Although YRS only accounts for 2.4% of the total area of the Yellow River Basin, its water yield accounts for 8.9% of the total runoff. 26% of the water in the Yellow River during the high and 40% in the low water period comes from the YRS. However, the research on WC in the Yellow River Basin often focused on the Three-River-Source, and Loess Plateau [[3], [4], [5], [6]]. There has not been a single study on the WC of the YRS. Due to environmental limitations, there is a great lack of available in-situ data in the YRS, so investigating the long-term changes of WC in this area extremely requires remote sensing data. In recent years, due to the influence of climate change and human activities, YRS has faced problems such as deterioration of the ecological environment, decreased WC, and increased water and soil loss [7]. In October 2021, the Chinese government proposed in the “Outline of the Yellow River Basin Ecological Protection and High-Quality Development Plan” to strengthen the WC capacity in the upper reaches of the Yellow River, and to promote wetland protection and ecological management in YRS [8].

WC is one of the important service functions of the ecosystem. The role of WC is manifested in the influence on the water cycle process, such as maintaining precipitation, inhibiting evaporation, regulating surface runoff, and regulating river discharge [9,10]. The evaluation of the regional WC function can be reflected by the WC amount [11]. With the development of ecosystem service models and computer technology, the evaluation of WC has been developed from field investigation to model calculation. Wang et al. [11] calculated the WC amount of Xiong'an New Area from 2007 to 2017 by combining the water balance method and the soil and water assessment tool (SWAT). They analyzed the spatio-temporal changes of WC amount using multiple linear regression and geographic detector. However, for long-term and large-scale studies, more efficient models are needed. Many studies evaluate WC using the Integrated Valuation of Ecosystem Services and Tradeoffs (InVEST) model. The spatio-temporal changes in water conservation in the Tibetan Plateau during 1961–2017 were analyzed by InVEST model and linear regression [12]. Based on InVEST model, the spatio-temporal water conservation dynamics in the Danjiang River Basin from 2000 to 2019 and its response to climate, land use and soil change were analyzed [13]. The water yield module of InVEST model is based on the Budyko water balance equation. Currently, the water balance method based on the InVEST model is the most widely used in the quantitative research of WC evaluation [12,14,15]. It takes the difference between precipitation and consumption as the WC [16]. However, the accuracy of InVEST has been questioned. The Z factor in the InVEST model is difficult to determine, leading to large uncertainties in WC results [17]. Moreover, many of the data used are not universal, such as root restricting layer depth, plant available water content, etc., which are unsuitable for long-term research [18]. GEE is a cloud platform for multi-source data and geospatial analysis [19]. GEE overcomes the limitations of the InVEST model in data selection by providing sufficient available data. Through the visual user interface and cloud computing, the adaptability and efficiency of the algorithm are improved [20,21].

LC is the main factor affecting WC, which humans can transform [22]. In 1999, the Chinese government launched a pilot program of “Grain for Green” (GFG) in Sichuan and other provinces, which was officially launched in 2002 [7]. GFG aims to convert farmland to forest or grassland and to increase local WC capacity by artificially altering LC. Cellular Automata Markov (CA-Markov) model retains the advantage of the Markov chain and enhances the simulation ability of spatial patterns through domain relationship analysis [23]. To better understand the regional dynamic changes, the CA-Markov model can simulate complex geographical processes [24,25]. LC changes in the Shadegan wetland was predicted using CA-Markov and supported the analysis of local water provision and habitat quality [18]. According to the LC change trend of the Halgurd-Sakran from 1993 to 2003 and 2003 to 2017, two scenarios were assumed to predict the 2023 LC change [26].

This study aims to evaluate and analyze WC changes in YRS and to provide solutions for improving local WC. The paper is organized as follows: (1) The WC in YRS from 2001 to 2020 was quantitatively evaluated by multi-source remote sensing data based on GEE. (2) The spatio-temporal variation patterns of WC during the study period were analyzed. WC and climate changes and their extreme changes, and the changes of LC and its WC in YRS during 2001–2020 were comprehensively presented. (3) The future development trend of WC and LC under the original scenario, GFG scenario, and current scenario were predicted by CA-Markov.

## 2 Materials and methods

### 2.1 Study area

**Fig. 1:**
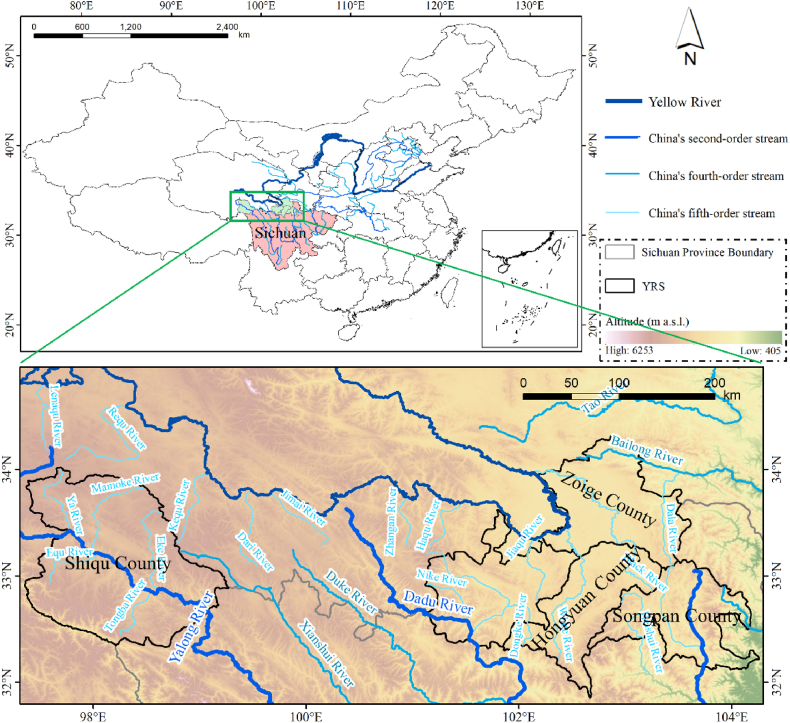
The location and landform of YRS.

YRS (97°21′–104°38′E, 33°33′–34°19′N) is located in the northern Sichuan Province, China, and consists of the whole region of Shiqu, Aba, Zoige, Hongyuan, and Songpan County (Fig. 1). In the study region, the mainstream of the Yellow River is 174 km long, with a drainage area of 18,700 km^2^. YRS is to the eastern edge of the Qinghai-Tibet Plateau, with an average altitude of over 3,500 m. The main tributaries of the Yellow River are the White River, Black River, and Jiaqu River, and the lower reaches are mostly valleys with flat terrain. YRS belongs to the plateau cold temperate monsoon climate, characterized by coldness, sufficient sunshine, large temperature difference between day and night, seasonless, long winter without summer, and annual permafrost time of about six months. The average yearly temperature is −1.1–5.7 °C, and the average annual precipitation is 570–861 mm, concentrated in the rainy season (May ∼ July). The oxygen content of YRS is only about 50% of that of Chengdu Plain. YRS has a long plant growth cycle, a fragile ecological environment, a large degradation area, desertification, and rodent infestation. The grassland and wetland ecology are easily damaged and difficult to restore [27,28].

### 2.2 Data

The Moderate Resolution Imaging Spectroradiometer (MODIS) series products were given priority due to their high resolution and data matching. The algorithm used for the MOD16A2.006 data collection is based on the Penman-Monteith equation [29], which includes inputs of daily meteorological reanalysis data along with MODIS remotely sensed data products such as vegetation property dynamics, albedo, and LC. The MCD12Q1.006 was derived using supervised classifications of MODIS Terra and Aqua reflectance data [30,31]. The supervised varieties then undergo additional post-processing incorporating prior knowledge and ancillary information to provide six classification schemes. In this study, we adopted the International Geosphere-Biosphere Programme (IGBP) global vegetation classification scheme [32,33], which contains 17 LC types and is one of the most widely used LC classification schemes. Land use/land cover data in China (LUCC) data with 30 m spatial resolution were used to validate the accuracy of MCD12Q1.006. The LUCC dataset is provided by the Data Center for Resources and Environmental Sciences, Chinese Academy of Sciences (RESDC). We randomly selected 1000 grids on the LC data in 2005,2010,2015, respectively, for validation with 90.13% accuracy.

The GEE platform provides multiple optional precipitation data. We used the Climate Hazards Group InfraRed Precipitation with Station data (CHIRPS). CHIRPS incorporates 0.05° resolution satellite imagery with in-situ station data, which is relatively suitable precipitation data for this study.

At present, there are only five runoff datasets available on GEE. ERA5, FLDAS, CFSR, and GLDAS were abandoned due to lower spatial resolution or limitations of data availability time. TerraClimate was generated by using climatically aided interpolation [34,35] to combine high-spatial resolution climatological normals from the WorldClim dataset [36] with coarser resolution time-varying data from CRU Ts4.0 and JRA55 [37,38]. WorldClim dataset is created by interpolating monthly average climate data from weather stations compiled from global, regional, national and local sources.

All the data used in this study are from GEE, and the data description is shown in Table 1. Only the data from the rainy season from May to July of each year were used for the WC calculation. The sum of each data in the rainy season is obtained by the “ee.ImageCollection.sum” function on the GEE. We unified the spatial resolution to 500 m for the data with different spatial resolutions. All the data were unified under the WGS 84 coordinate system. The “Export.image.toDrive” function on the GEE accomplished the resolution unification and assignment of coordinate systems. The number of grids calculates the area, and the area of each grid is 0.25 km^2^.

**Table 1:** Data used from GEE.

Data	Data source	Spatial resolution	Temporal resolution
Precipitation	CHIRPS [39]	0.05°	Daily
Evapotranspiration	MOD16A2.006 [40]	500 m	Monthly
Runoff	TerraClimate [41]	0.04°	8-day
Land cover	MCD12Q1.006 [42]	500 m	Yearly

### 2.3 Framework

**Fig. 2:**
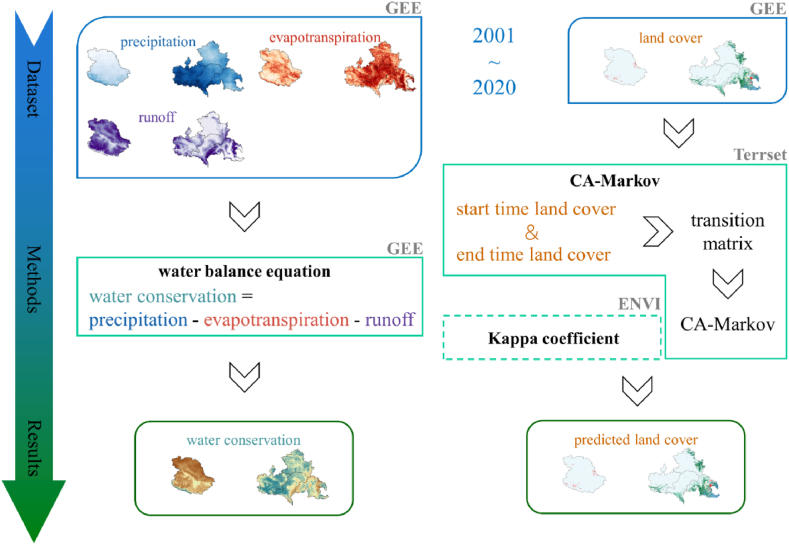
Flowchart of this study.

The specific process of this research is shown in Fig. 2, which is divided into three parts. 1) Data preparation: including precipitation (PR), evapotranspiration (ET), runoff (RO), and LC data. The data sources and resolutions used in this study are listed in Table 1. 2) Methods: WC was calculated quantitatively by the water balance equation, and CA-Markov was used to predict LC changes. 3) Results and analysis: Results were presented in clear graphs and tables. CA-Markov predicted future LC and WC changes. Finally, the conclusions and suggestions of this study were put forward.

### 2.4 Water balance equation

The water balance equation of WC is constructed based on the Budkyo curve, which regards WC as the difference between PR and RO [43]. If the equation takes zero, the water gains and losses are equivalent, and WC is unchanged. We defined the WC calculation method by combining the approaches suggested in the InVEST [44], SWAT [45] and WEP-L methods. Most researchers have adopted the water balance equation. The difference is that ET and RO are calculated by different models [11,46,47]. The WC calculation equation (Equation (1)) in this study is as follows:

(1) WC=PR−ET−RO

where WC is the water conservation (mm), and PR, ET, and RO are precipitation, evapotranspiration, and runoff, respectively (mm). The values of all variables in the equation are the sum of May, June, and July and represent annual values. Among them, the unit of ET (kg/m^2^) is different from others (mm), but it is equivalent. In this study, the unit of these will be unified into (mm), and the regional total will be counted in (m^3^), to be consistent with previous research [13,48]. The equation is fully implemented with GEE through “ee.Image”.

### 2.5 CA-Markov model

CA-Markov was proposed by Clark Labs in 1987 and implemented by the IDRISI analysis module in TerrSet software [49]. CA-Markov integrates the Markov chain, Cellular Automata, and Multi-Criteria/Multi-Objective Land Allocation (MOLA), and has more advantages in the simulation and predicting spatial changes [50].

Markov chain is a set of discrete random variables with Markov properties and is the crucial transition matrix in CA-Markov. The Markov chain is obtained through the initial and end states of land changes and is implemented through the Markov function in IDRISI, the transition matrix (Equation (2)) is as follows:

(2) $$P_{ij}=\left[\begin{array}{ccc} P_{11} & \cdots & P_{1n}\\ \vdots & \cdots & \vdots \\ P_{n1} & \cdots & P_{nn} \end{array}\right]$$

where P_ij_ is the transition matrix, n is the number of LCs, and two conditions require: $0\leq P_{ij}\leq 1;\;\sum_{j=1}^{n}P_{ij}=1$.

Cellular Automata is a grid dynamics model in which time, space, and state are discrete, and space interaction and time causality are local. CA-Markov has the characteristics of Cellular Automata, which endow it with the ability to simulate the temporal and spatial evolution of complex systems. CA-Markov model can be defined as Equation (3) [51]:

(3) S(t,t+1)=f(s(t),N)

where S is a set of cellular states, (t, t+1) represents the beginning and ending time, N is the cellular neighborhood, and f is the cellular local transformation rule.

The specific implementation steps and settings of CA-Markov are as follows:

1. Transition matrix: Calculation of transition matrix according to LC change between the beginning and ending time. According to user help and previous research [52], setting the background cell option to “Assign equal probabilities” and the proportional error to “0.15”. However, the result under this setting is not ideal, and the predicted LC shows a mosaic effect. After constantly adjusting the parameters, we finally set the background cell option to “Assign 0.0” and the proportional error to “0.0”, which can get more accurate results;
2. CA-Markov: The standard 5 × 5 contiguity filter is applied. The number of Cellular Automata iterations needs to be determined based on the time interval, and one iteration represents one year;
3. Accuracy assessment: As a classic image consistency test tool, Kappa is used to verify the prediction accuracy of CA-Markov [53]. The Kappa coefficient was calculated using the confusion matrix tool provided by ENVI software, and the formula is as follows (Equation (4)):

(4) $$\begin{array}{c} Kappa=\frac{\left(P_{0}-P_{e}\right)}{\left(1-P_{e}\right)}\# \end{array}$$

where P_0_ is the sum of the number of correctly predicted pixels in each LC divided by the total number of pixels, and P_e_ is the sum of the product of the actual pixels and the correctly predicted pixels in all LCs.

### 2.6 Abrupt change point detection

This study investigated long-term climate and WC changes, therefore involves analysis of abrupt change point at different phases or extreme situations. The Mann-Kendall Test [54,55] is a non-parametric test that is one of the widely used method for detecting trends in the time series. The Mann-Kendall test can determine whether there are mutations in sequence data, and the specific formula is shown in Equations (5)–(7):

(5) $$\begin{array}{c} d_{k}=\sum_{i=1}^{k}m_{i}\# \end{array}$$

where m_i_ denotes the value of the i-th sample (1 < k ≤ i) in the series data (i = 1, 2, 3, …, n). Under the assumption that the original sequence is randomly independent, the mean var (d_k_) and variance UF_k_ are:

(6) $$\begin{array}{c} var\left(d_{k}\right)=k\;\left(k-1\right)\left(2k+5\right)/72\# \end{array}$$

d_k_ is normalized to obtain UF_k_:

(7) $$\begin{array}{c} {UF}_{k}=\left(d_{k}-E\left(d_{k}\right)\right)/\sqrt{var\left(d_{k}\right)}\# \end{array}$$

Repeat the above process in reverse chronological order, while making UB_k_ = -UF_k_, k = n, n-1, …, 1), UB_1_ = 0. If the intersection point of the UF_k_ and UB_k_ curves lies within the confidence interval, then the intersection corresponds to the time when the mutation occurred.

## 3 Results

### 3.1 Variation of water conservation and climate factors

**Fig. 3:**
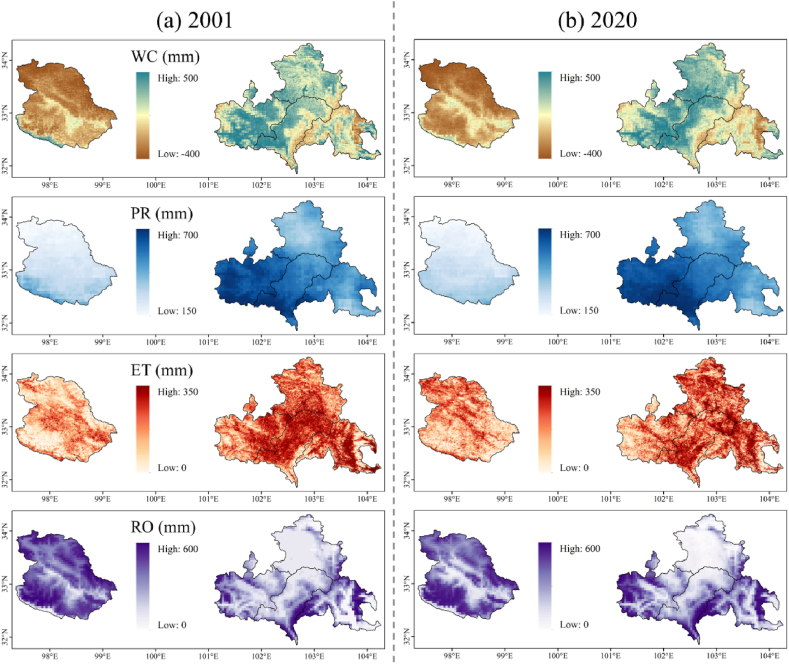
Spatial distribution of WC and climate factors in 2001 and 2020.

According to the principle described in Section 2, the spatial distribution of WC in the YRS from 2001 to 2020 was calculated. Fig. 3 (a) and (b) shows the spatial distribution of WC in 2001 and 2020. YRS can be divided into Shiqu County and the eastern four counties (EYRS) according to the difference in geographical location and result characteristics. Shiqu County has lower PR and ET but higher RO, which results in lower WC. This phenomenon may be due to its underlying surface conditions, such as terrain (higher altitude) and landforms (LC types with lower WC capacity). For EYRS, the spatial distribution of the results has some visible regularities (Fig. 5 (a)). EYRS has higher ET, and its southern edge region has higher RO. In EYRS, PR has a decreasing trend from west to east, and WC has a similar tendency. Furthermore, the spatial distribution of results has a high correlation with elevation (Fig. 1). Areas with lower elevation generally have higher WC, ET, and lower RO, and vice versa.

**Fig. 4:**
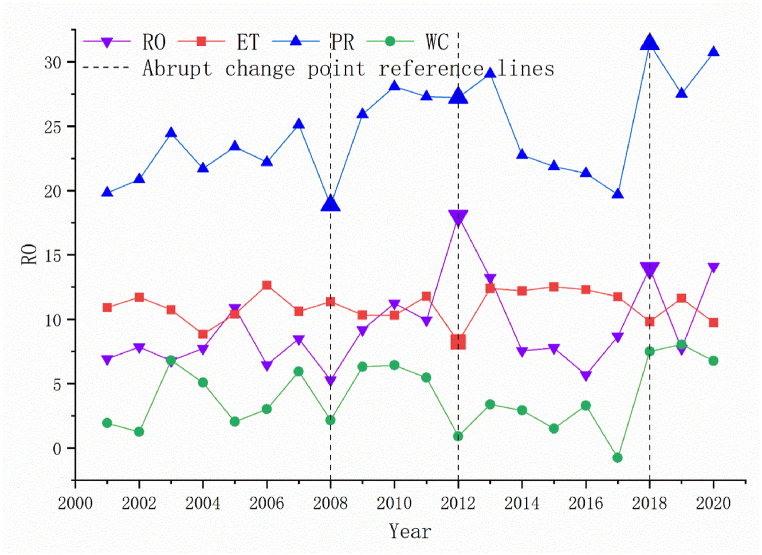
Changes in WC and climate factors from 2001 to 2020.

From 2001 to 2020, the annual WC in YRS showed a fluctuating increase trend, with an average increase of 4 billion m^3^, and finally, increased from 1.93 to 6.77 billion m^3^ (Fig. 4). The annual WC varied from −0.76 billion to 7.49 billion m^3^, with the minimum and maximum values appearing in 2017 and 2018, respectively. The continuous decline of PR from 2014 to 2017 may lead to a decrease in WC and the occurrence of minimum WC. PR, ET, and RO average values are 24.46, 11.02, and 9.37 billion m^3^, respectively. Among them, PR has the highest randomness, followed by RO and ET, with 13.53, 10.30, and 1.46, respectively. Due to the largest volume and randomness of PR, PR plays a dominant role in the variation of WC, showing similar inter-annual variation trends to WC. In addition, sudden changes in RO greatly impacted on WC, especially in 2012.

**Fig. 5:**
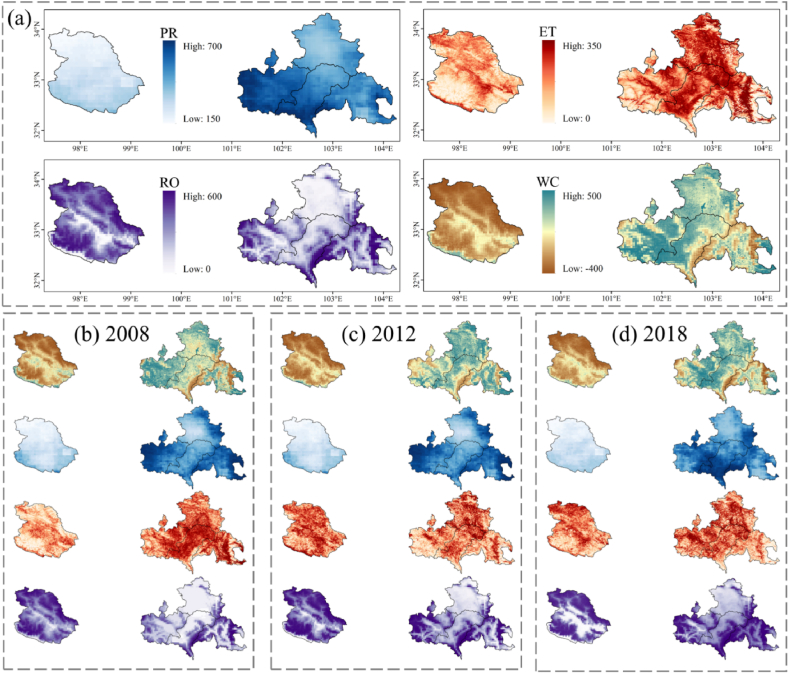
Spatial distribution of extreme climate factors and corresponding WC changes. (a) The mean values of climate factors and WC from 2001 to 2020. (b) ∼ (d) correspond to the spatial distribution in 2008, 2012 and 2018, respectively. And the legend and location information for all subplots are consistent with (a).

There are many unusual changes in the curve of Fig. 4, and the corresponding extreme climate factors and WC are shown in Fig. 5. The abrupt change points identified by the Mann-Kendall test occurred in 2008 (Fig. 5 (b)), 2012 (Fig. 5 (c)), and 2018 (Fig. 5 (d)), and the corresponding symbol of the climate factors has been increased (Fig. 4). In the spatial distribution, no abrupt change points were detected in the change of total WC, and only the transitions of WC in Hongyuan and Songpan counties were detected in 2018. Consistent with the conclusion in the previous paragraph, PR is the main factor affecting the abnormal change of WC. Distinguishingly, in 2012, the only year not dominated by PR, the higher ET and RO in Shiqu County contributed to the low WC. This is because changes in RO especially occur in areas with low RO, while areas with higher RO are more stable. And the distribution difference of PR is mainly in Aba, Hongyuan, and Songpan County in EYRS. Therefore, with lower RO, Aba, Hongyuan, and Songpan County are more vulnerable to extreme climates.

**Fig. 6:**
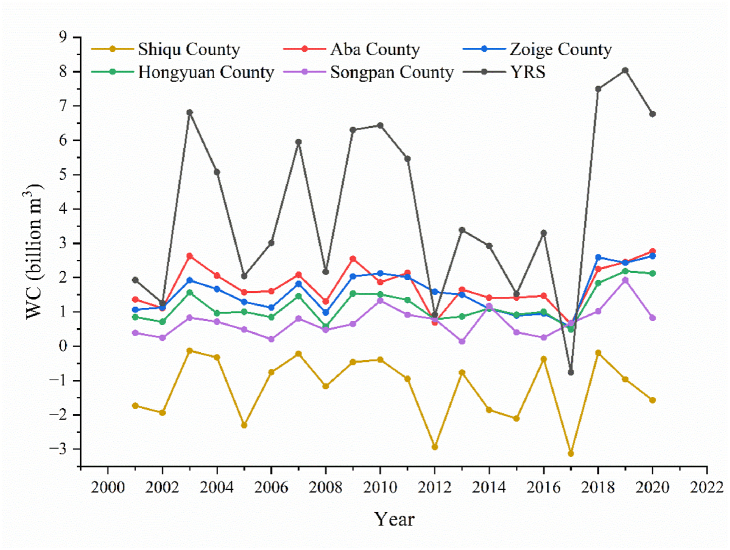
Changes of WC in different counties from 2001 to 2020.

The order of WC from high to low is: Aba, Zoige, Hongyuan, Songpan, and Shiqu County, with an average of 1.75, 1.57, 1.18, 0.71, −1.21 billion m^3^, respectively (Fig. 6). From 2001 to 2020, the improvement of WC of each county from high to low is: Zoige, Aba, Hongyuan, Songpan, and Shiqu County, with increased volumes, are 1.57, 1.40, 1.27, 0.43, and 0.16 billion m^3^ respectively. In general, the WC of the counties in EYRS is similar, and the difference in the total volume of WC is mainly due to the difference in the county area. Uniquely, Shiqu County has a negative annual WC every year, which means continuous loss of water resources and a greater risk of soil erosion. Although Shiqu County has the lowest PR and ET, it has the highest RO, accounting for about 54.9% of the YRS. As shown in Fig. 1, Fig. 3, higher elevation may have resulted in higher RO, indicating that terrain may have determined WC in Shiqu County.

### 3.2 Variation of land cover and its influence on water conservation

**Fig. 7:**
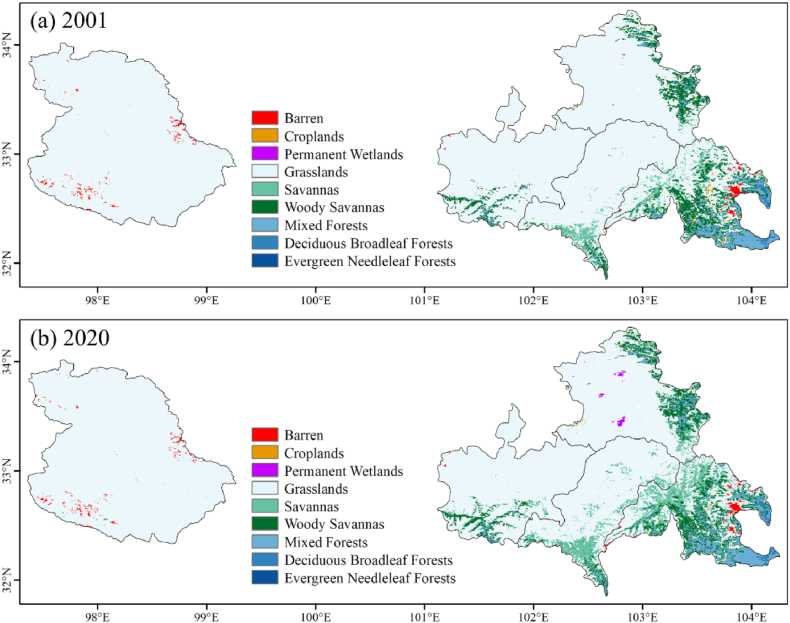
Spatial distribution of LCs in 2001 and 2020.

**Fig. 8:**
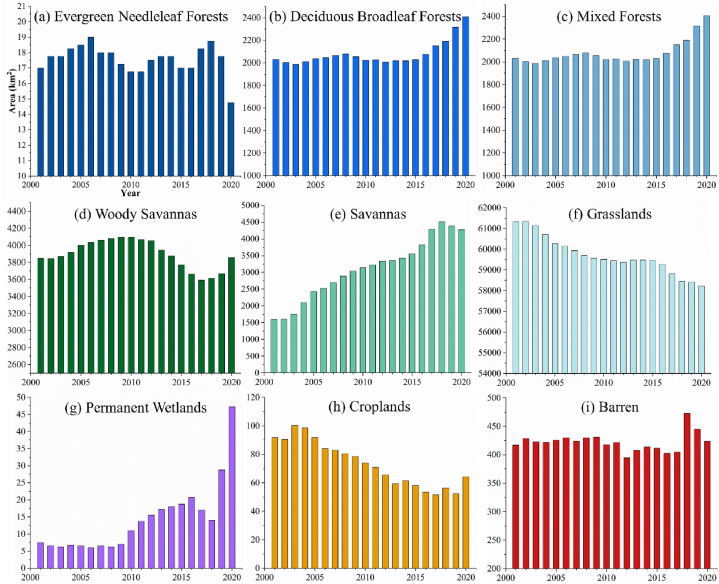
Area (km^2^) change of different LC.

The nine LC types in YRS are shown in Fig. 7 (a) and (b). Other LC types (water, ice and snow, urban and built-up lands, and open shrublands) were excluded due to their small area (less than 0.005%). The majority of LC type in YRS is grassland, about 60,000 km^2^, accounting for 88.1% of the total area, as shown in Fig. 8 (f). The second is the woody savannas (5.53%) (Fig. 8 (d)), followed by mixed forest (2.92%) (Fig. 8 (c)) and savannas (2.29%) (Fig. 8 (e)), mainly distributed in Songpan County, eastern Zoige County, and the southern edge of YRS. The forests are mainly located in the southeast of Songpan County, with deciduous broadleaf forests (Fig. 8 (b)) accounting for 0.379% and evergreen needleleaf forests (Fig. 8 (a)) accounting for 0.024%. Almost all wetlands are located in Zoige County (0.011% of the total area of YRS), and the wetland area has increased significantly between 2001 and 2020 (Fig. 8 (g)). Most of the cropland is in Songpan County (0.132% of the total area of YRS), which is gradually replaced by grass and forests (Fig. 8 (h)). The Barren is mainly in eastern Songpan County and Shiqu County (0.599% of the total area of YRS), with little change during the study period (Fig. 8 (i)).

The GFG policy has been fully implemented since 2002 [7], and it is an important influencing factor in LC-type changes. Correspondingly, the area of croplands and grasslands has decreased significantly, while the size of wetlands and savannas has increased rapidly in nearly two decades. Since 2003, cropland area decreased by 27.5 km^2^ with a change rate of −30%, and grassland had the largest reduction in size and the smallest reduction rate, which are 3101.8 km^2^ and -5.1%. On the other hand, the wetland area increased by 39.8 km^2^ with a change rate of +530%, especially in recent years. And savanna area increased by 2678.8 km^2^ with a change rate of +168%. In addition, the area of deciduous broadleaf and mixed forests is also growing, and the area of deciduous broadleaf forests and woody savannas decreased after increasing.

**Fig. 9:**
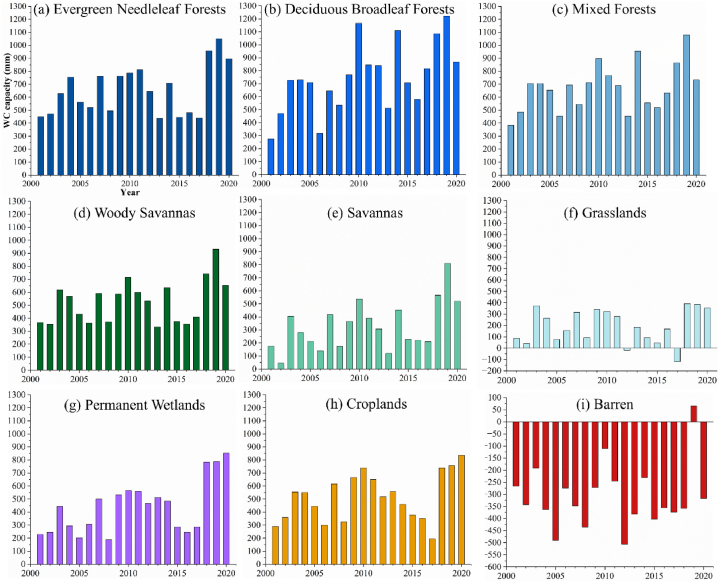
WC capacity per km^2^ (mm) change of different LC.

Previous studies have shown that WC is correlated with LC [53,56]. To better understand the WC capacity of different LC under different climate and environmental conditions, the WC of each LC during the study period is summarized in Fig. 9 (a) – (i). The WC capacity of each LC is the average WC on all its grids. Reasonably, forests have the highest WC capacity (e.g., deciduous broadleaf forests), and barren has the worst WC capacity. Except for the barren, all LC show different sensitivities to climate, which are manifested as periodic changes, and this trend has not changed completely. Wetlands exhibit higher PR sensitivity. Unexpectedly, croplands contribute a higher WC, while grasslands have a low WC, almost contrary to the original intent of the GFG policy. This may be because croplands are usually situated in a good environment with higher WC, while grasslands have always maintained their original environment, which makes it difficult for trees to grow and has poor WC capacity.

### 3.3 Simulating and forecasting the changes in LC and WC

CA-Markov is used to predict future LC changes. On this basis, the available capacity of each LC can be used to predict future WC changes. First, we tested the CA-Markov prediction ability by the Kappa coefficient. Next, considering the impact of climate and policy, we will divide into three scenarios to speculate on the prospects of YRS:

4. Original scenario: From 2001 to 2002. Before the GFG policy, there may be overgrazing and unreasonable land use;
5. GFG scenario: From 2003 to 2020. Since GFG policy was fully launched and aims to explore the benefits of continuous implementation of GFG policy;
6. Current scenario: From 2018 to 2020. It is characterized by increased PR, and the area of LCs with many high WC increases again (Figs. 4 and 8).
7. Real 2020: We used the real 2020 WC and LC as a control group to reflect the projected changes by 2025.

#### 3.3.1 Accuracy assessment of CA-Markov

To examine the performance of CA-Markov over a larger period and under different climatic conditions (Fig. 3), we selected the period from 2005 to 2015 for validation. Then the transition matrix from the LC change from 2005 to 2010 was calculated and predicted the LC in 2015 based on the LC in 2010. The Kappa coefficient is obtained by calculating the confusion matrix between the predicted LC (CA-Markov) with the real LC. The output confusion matrix is shown in Table 2.

**Table 2:** The confusion matrix between the predicted LC with the real LC in 2015 (unit: pixels/0.25 km^2^).

Real Predicted	Evergreen Needleleaf Forests	Deciduous Broadleaf Forests	Mixed Forests	Woody Savannas	Savannas	Grasslands	Permanent Wetlands	Croplands	Barren	Total
Evergreen Needleleaf Forests	54	0	4	0	0	0	0	0	0	58
Deciduous Broadleaf Forests	0	884	55	86	183	3	0	0	0	1211
Mixed Forests	18	84	7369	626	7	11	0	0	0	8115
Woody Savannas	3	13	1164	12827	2009	987	7	0	0	17010
Savannas	0	5	7	165	12148	5336	0	0	14	17675
Grasslands	0	2	9	571	3645	225244	49	113	430	230088
Permanent Wetlands	0	0	3	3	1	4	50	0	17	79
Croplands	0	0	0	0	0	108	0	108	0	216
Barren	0	0	0	0	0	176	1	0	1358	1540
Total	75	988	8611	14278	17993	231870	107	221	1820	

The output shows the overall accuracy is 98.1%, and the Kappa coefficient reaches 0.96, which indicates that CA-Markov is reliable in predicting LC changes in YRS. The accuracy of LC prediction from high to low is: for grasslands, mixed forests, woody savannas, evergreen needleleaf forests, barren, deciduous broadleaf forests, savannas, permanent wetlands and croplands, and the accuracy rate is from 49.4% to 97.5%. Only the accuracy of savanna, cropland and permanent wetlands is lower than 80%, and the grasslands and forests with the largest area have high prediction accuracy. The wrong prediction of cropland and wetland may stem from their small area and frequent transformation. Grass and tree mispredictions are often in grasslands, and wrong predictions also tend to overestimate the density and height of vegetation.

#### 3.3.2 Prediction of future changes of LC and WC in YRS

**Fig. 10:**
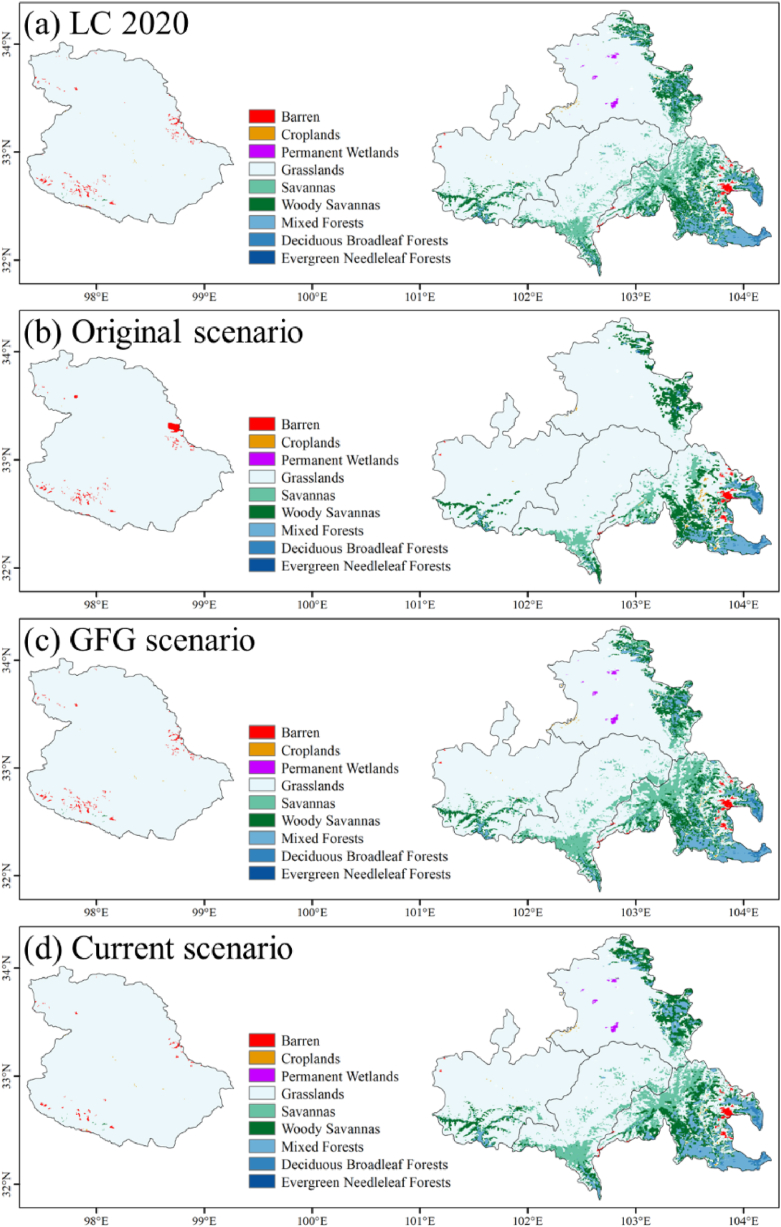
Comparison between the real LC in 2020 and the predicted LC in 2025 under different scenarios.

We used CA-Markov to predict 2025 LC through the LC changes of the starting and ending dates with different scenarios. Fig. 10 shows the predicted LC changes to 2025 and the real LC in 2020 (Fig. 10 (a)) as a comparison. Under the original scenario (Fig. 10 (b)), wetlands and savannas may be severely degraded, while croplands and barren in Shiqu County may increase, which means lower WC capacity in the future (Fig. 9). The difference between the GFG (Fig. 10 (c)) and the current scenario (Fig. 10 (d)) is mainly reflected in the conversion of grass and woodland. The current scenario may include more deciduous broadleaf forests, mixed forests, and woody savannas (Table 3). And the barren area in Shiqu County will decrease under the current scenario, which has not happened in the past 20 years. Moreover, the area of evergreen needleleaf forests increased under the original scenario, which may indicate that YRS is no longer suitable for evergreen needleleaf forest growth due to the changes in climate and environment.

**Table 3:** Prediction of LC under different scenarios in 2025.

Scenarios LC (km^2^)	Real 2020	Original	GFG	Current
Evergreen Needleleaf Forests	14.8	28.8	13.3	8.3
Deciduous Broadleaf Forests	285.3	287.3	269.3	307.5
Mixed Forests	2407.3	1493.2	2482.5	2917.5
Woody Savannas	3856.5	3592.5	3745	4246
Savannas	4272.8	1707.75	4855.8	3859.2
Grasslands	58217	61199.2	56981.7	57117.3
Permanent Wetlands	47.3	3	44.5	37.8
Croplands	64.3	78.5	57.25	56.3
Barren	424	452.7	390.7	292.5

To assess future WC changes, we used the local 20-year average WC of each LC to predict the WC of YRS in 2025 (Table 4). The predicted WC is the product of each LC type's indicated area and average WC capacity. Without the GFG policy, WC will remain at the level in 2000, with a reduction of about 4.53 billion m^3^. In the future, GFG policy is expected to contribute 0.76 billion m^3^ of WC, with an average yearly increase of 0.15 billion m^3^. Compared with the average annual WC growth (0.24 billion m^3^) in the past 20 years, the improvement of WC by GFG policy will gradually decrease. The climate will still be the main factor affecting WC in the future. Under the current scenario, WC is expected to increase by 1.7% by 2025, equivalent to the gains of the past 11 years.

**Table 4:** Prediction of WC under different scenarios in 2025.

Scenarios LC (billion m^3^)	Real 2020	Original	GFG	Current
Shiqu County	−1.58	−1.96	−1.08	−0.37
Hongyuan County	2.12	0.92	1.94	2.11
Songpan County	0.82	0.46	1.09	1.17
Aba County	2.77	2.01	2.78	2.33
Zoige County	2.63	0.81	2.82	3.23
Total	6.77	2.24	7.54	8.47

Hongyuan and Aba County may have WC losses in the future, with −0.01 and −0.44 billion m^3^ in the current scenario and −0.18 and 0.01 billion m^3^ in the GFG scenario. On the other hand, WC in Songpan and Zoige County will keep growing in the future, with 0.34 and 0.6 billion m^3^ in the current scenario and 0.27 and 0.19 billion m^3^ in the GFG scenario. Zoige County has the largest difference in WC capability under different scenarios, which may contribute 0.5 to 1.21 billion m^3^ of the WC. This difference comes from the changes in forests and wetlands in Zoige County (Table 3). Forests have high WC capacity, while wetlands have no direct WC contribution, but it can improve the environment to allow tall WC plants to grow (Fig. 9). Moreover, if the wetland is degraded into grassland, there will be a large WC loss. After that, Shiqu County also shows the difference in future WC under different scenarios. The GFG policy has not improved the WC situation in Shiqu County, and the WC capacity of Shiqu County is still insufficient to deal with climate change. As in the past two decades, the other three counties have similar WC capabilities under different scenarios.

## 4 Discussion

### 4.1 Optimization of the method

In this study, We introduced GEE to evaluate WC in YRS during 2001–2020. Due to the limitation of environmental conditions in the YRS, the extensive use of remote sensing data is essential. InVEST model was the first model we considered using due to the high correspondence between its required data and the available remote sensing data. But in practice, there were many insurmountable obstacles. Firstly, WC is obtained by adding correction factors such as RO to the water yield calculated by InVEST, and its applicability has not been verified [13,57]. Second, the variable water yield is derived from some immutable reference data [[58], [59], [60]]. After that, the empirical coefficient Z factor, which cannot be precisely determined, introduces a large bias in the WC evaluation [17]. In addition, the actual ET and RO are usually calculated by the model in the studies of WC evaluation based on the water balance method [11,14,61], which may affect the confidence of the results. Therefore, we chose public datasets (such as CHIRPS and TerraClimate) to replace the intermediate process of model calculation, with higher resolution, real-time, and reliability. Furthermore, these datasets can be easily obtained from GEE, which means this method can be fully replicated in later studies. Compared with traditional methods, the advantages of the WC calculation based on GEE lie in a more transparent theoretical basis, lower time cost, and more reliable data selection [62].

CA-Markov was used to predict the LC change in 2025. The performance of CA-Markov has been demonstrated in many researches, and the Kappa coefficient in different regions exceeds 0.8 [47,63,64]. Although many studies used CA-Markov to predict LC changes in the future over ten or even 30 years [65,66], we only predicted changes in the next five years. The aim is to ensure the accuracy of the prediction, and to compare the development trend of LC under different scenarios. In addition, we forecasted WC in 2025 based on the predicted 2025 LC and its corresponding average WC capacity over the past two decades. Compared with previous studies, Zeng et al. [61] combined the bayesian belief network and CA-Markov to predict the WC pattern of the Weihe River through the LC change, but the WC was not quantified. Without CA-Markov or other methods, most WC researchers did not report the prediction of future WC [14,22,48].

### 4.2 Analysis and proposal of water conservation pattern

YRS is located on the eastern edge of the Qinghai-Tibet Plateau. Its alpine ecosystem is easily damaged and difficult to restore. Meanwhile, YRS belongs to the source area of the Yellow River, and its WC capacity greatly impacts on the downstream soil erosion and ecological environment. However, there has been no research on the WC of the YRS. The WC in YRS has gradually increased since 2003, which is contrary to the trend before 2003 [3]. Consistent with relevant studies on the Qinghai-Tibet Plateau, the spatial distribution of WC in YRS decreased from east to west due to the difference in climate and vegetation coverage [12,67]. Climate change is considered to be the direct cause of WC change. Consistent with previous studies, we found a high correlation between PR and WC [46,48]. RO is a quantitative indicator in this study, so we found a high correlation between RO and WC in spatial distribution. And through the similarity of the spatial distribution of elevation and RO, we determined that RO partly reflects the correlation between elevation and slope and WC. During the study period, changes in RO were usually determined by PR and affected WC. However, it is unclear why RO and ET changed significantly with stable PR in 2012, and there was no relevant record.

The environmental deterioration in YRS is mainly due to human factors [68]. We reflected on the impact of human activities on YRS through LC changes. Improved over previous WC studies, the statistics of LC change and WC capacity in our research are explicit on the annual-scale [46,69]. Many studies have demonstrated the high WC capacity of forest and grassland, increasing WC capacity with vegetation coverage and height [13,70]. However, in YRS, grassland has poor WC capacity, while cropland has high WC capacity, so the local GFG policy may need to be transformed. Cropland only accounts for 0.13% of the total area but has decreased by 30% in the past two decades. In particular, cropland has no obvious negative impact on WC. Therefore, we believe blindly implementing the GFG policy will not bring about significant WC improvement but will cause land waste and economic losses. And grassland and barren areas with large and low WC should be more concerned. Furthermore, the natural wetlands in Zoige County have huge WC potential, and their restoration should be promoted [28,71].

In fact, the ecological functions of each section of the Yellow River Basin are different, and the corresponding policies should be implemented according to local conditions. Lv et al. proposed that the LC transformation from bare land to grassland or farmland to forest land can significantly reduce both RO and sediment transport in the Upper Yellow River Basin [72]. For the EYRS region, a comprehensive grazing ban or regional rotational grazing should be implemented [73]; while in Zoige County, planting grasslands to reconstruct wetlands should be an efficient method for ecosystem restoration [28]. The challenges of inefficient GFG policy and climate change will have to be faced in the future. With the convening of relevant meetings, the ecological governance of YRS has entered a new stage, and now is the chance to promote policy transformation. As the first stop of the Yellow River entering YRS, Shiqu County has the primary WC responsibility in YRS. On the contrary, Shiqu County has the lowest WC capacity, and there are large areas of grassland and barren. Considering the lower PR and topography of Shiqu County, it may be a good choice to reduce the barren area, increase the grassland coverage, and convert part of the grasslands into Savannas. In EYRS, converting grasses into mixed forests and forests is most important. After that, they are improving other LCs quality rather than reducing cropland.

### 4.3 Limitations and prospects

• WC evaluation method on GEE needs to be improved: Since there is no more hydrological mechanism to support, our method only ensures the feasibility at the watershed scale. It is worth looking forward to the implementation of complex physical models on GEE, which will promote the intelligence of hydrological research;
• Longer study period and higher spatio-temporal resolution: Due to the limitation of MODIS series products' availability time (2001-present), we did not extend our research to the years before 2000. For longer-period studies, the main challenge is matching the data. Low resolution for LC data caused mixed pixel problems, resulting in many LC types not being well differentiated. Higher resolution means higher precision, but there may be other problems. E.g., Dynamic World is a 10 m near-real-time LC data provided by Google, but the data is only available after 2015. Furthermore, since the climate in YRS is not significant except for the rainy season, we neglected the WC change within a year. Nevertheless, seasonal WC changes are more reflective of local WC function;
• Validation of WC evaluation: According to the government report, the annual average water resource volume of YRS is 4.4 billion m^3^ [74], which is close to the average WC in this study (4 billion m^3^). At present, there is still no unified standard for the evaluation of WC, and the methods for WC evaluation are very diverse (including SWAT and InVEST, etc.) [[75], [76], [77], [78], [79], [80]]. Existing researches usually reflect WC function/capacity in terms of WC amount, and none have verified the evaluation results [[10], [48], [70], [81], [82], [83]]. The reason may be the regional differences in relevant studies and the lack of validation data. Unified WC evaluation standards and validation have yet to be reached.

## 5 Conclusions

This study evaluated water conservation in the Yellow River Basin in Sichuan Province from 2001 to 2020 and predicted land cover change and water conservation in 2025 under three scenarios based on GEE and CA-Markov. The total WC of YRS increased from 1.93 to 6.77 billion m^3^. Climate change is the main cause of WC changes in YRS. Precipitation dominated WC's change, runoff affected WC's spatial distribution, and evapotranspiration was relatively stable. Extreme climate change is more likely to cause abnormal WC in Aba, Hongyuan, and Songpan County. Due to the difference in climate and environment, Aba, Zoige, Hongyuan, Songpan, and Shiqu County have similar annual WC of around 1.30 billion m^3^; but Shiqu County has been experiencing an annual average water loss of 1.21 billion m^3^. Although climate change determines WC, LC indirectly affects WC by reducing runoff and evapotranspiration. The WC decreases with the decrease in vegetation height and coverage. The forest has the highest WC capacity of 691.27 mm per km^2^, while that of barren is the lowest with −309.98 mm per km^2^. Unexpectedly, the cropland has a higher WC capacity of 514.21 mm per km^2^, but the grass is poorer of 191.59 mm per km^2^. By comparing the predicted WC under different scenarios, it is concluded that GFG contributes 4.53 billion m^3^ WC to the YRS (three times more than initially), but this benefit will gradually decrease, improving WC by only 0.77 billion m^3^ in the next 5 years. In the future, WC will continue to increase by no more than 1.70 billion m^3^, so local ecological management and policies need to be adjusted urgently. More attention should be paid to converting grasslands to forests rather than reducing cropland.

## Declarations

### Author contribution statement

Zhichong Yang: Performed the experiments; Analyzed and interpreted the data; Wrote the paper.

Xiaoai Dai: Conceived and designed the experiments; Analyzed and interpreted the data.

Heng Lu: Conceived and designed the experiments; Analyzed and interpreted the data; Wrote the paper.

Chao Liu: Conceived and designed the experiments.

Ruihua Nie; Min Zhang; Lei Ma; Naiwen Li: Contributed reagents, materials, analysis tools or data.

Tiegang Liu; Yuxin He; Zhengli Yang: Analyzed and interpreted the data.

Ge Qu; Weile Li; Youlin Wang: Performed the experiments.

### Data availability statement

The water conservation evaluation in this study is free available online at https://code.earthengine.google.com/?scriptPath=users%2Fsincerapr47%2FWater%3Awater%20conservation.

## Declaration of competing interest

The authors declare that they have no known competing financial interests or personal relationships that could have appeared to influence the work reported in this paper.
